# Neuronal signaling and behavior

**DOI:** 10.3389/fnbeh.2012.00072

**Published:** 2012-11-02

**Authors:** Gilberto Fisone, Riccardo Brambilla

**Affiliations:** ^1^Department of Neuroscience, Karolinska InstitutetStockholm, Sweden; ^2^Institute of Experimental Neurology, Division of Neuroscience, San Raffaele Scientific Institute and UniversityMilano, Italy; ^3^School of Biosciences, Cardiff UniversityCardiff, UK

The last years have witnessed a number of important technical breakthroughs, which have improved considerably the ability to investigate the contribution of molecular processes to physiological and pathological conditions. This is particularly evident with regard to the study of the involvement of specific signal transduction mechanisms in brain function and disease. Increasingly sophisticated transgenic approaches combined with the use of a vast array of behavioral models have led to a profound and detailed view of the way in which distinct brain regions and neuronal populations are affected by environmental and pathological challenges.

The scope of the present volume is to provide a panoramic view of recent progress in this direction, with special emphasis on key molecular mechanisms implicated in learning and memory function, as well as in neurodegenerative and neuropsychiatric disorders, including Parkinson's disease, epilepsy, schizophrenia, and drug addiction.

In the first Original Article, Penke et al. (2011) describe the existence of distinct mechanisms at the basis of the regulation, in the hippocampus, of *Arc/Arg3.1*, an immediate early gene implicated in synaptic plasticity. They show that, during spatial exploration, early expression of *Arc/Arg3.1* occurs independently of the transcription factor *Egr1/Zif268*. In contrast, *Egr1/Zif268* is necessary for a late, prolonged wave of *Arc/Arg3.1* expression in response to electroconvulsive shock.

Signaling in epileptic behavior is the subject of the Review Article by Bozzi et al. (2011). Here, the authors concentrate on the pathways activated by modulatory neurotransmitters (e.g., dopamine, norepinephrine, and serotonin), which lead to changes in gene expression during acute seizure events. They also discuss the deleterious consequences of seizure activity, focusing on the contribution of specific signaling pathways to the progression of the disease.

The Original Article by Pozzi et al. (2011) provides evidence indicating that activation of the cAMP response element-binding protein (CREB), at different locations within the corticostriatal circuitry, is associated to distinct behavioral responses involved in visuospatial attention.

Several Review Articles are centered on specific molecular mechanisms implicated in memory formation and cognition. Sindreu and Storm (2011) provide a timely and exhaustive overview of the importance of zinc homeostasis and zinc-activated signal transduction pathways [such as the extracellular signal-regulated kinase (ERK) cascade], in synaptic plasticity and memory, with special focus on the relevance of these mechanisms in the hippocampus and amygdala. On the same line, a comprehensive Review Article by Fasano and Brambilla (2011) deals with the implication of abnormal Ras-ERK signaling in drug addiction, intellectual disability and L-DOPA induced dyskinesia, a severe and frequent motor disorder affecting Parkinson's disease patients.

Two Original Articles by d*Isa et al. (2011) and Sindreu and Storm (2011), discuss the role of Ras-GRF1, a neuronal specific activator of Ras, in various forms of memory. Interestingly, Ras-GRF1 seems to have a complex role, being dispensable for most forms of declarative learning (but not object recognition), while playing a crucial role in emotional memories.

In their Review Article, Gal-Ben-Ari and Rosenblum (2012) discuss the behavioral paradigms to study the sense of taste with regard to learning, memory, and consolidation. They also present a comprehensive overview of the neuronal structures, the neurotransmitter systems, and the signaling components involved in these processes.

The application of transgenic techniques to link distinct neuronal populations and signal transduction pathways to specific behaviors has been particularly useful in the study of the basal ganglia, a group of brain structures affected by numerous neurodegenerative and neuropsychiatric diseases.

The Review Article by Ena et al. (2011) describes the contribution of new methodologies, such as bacterial artificial chromosome (BAC) transgenesis, optogenetic, and viral transgenesis, to the study of the striatum, which is the major component of the basal ganglia. The use of cell-specific ablation and conditional knock-out is discussed with regard to the characterization of the role played by distinct populations of striatal neurons in motor behavior and in motivational processes implicated in drug addiction.

Basal ganglia transmission and striatal neurons is also the main topic of the Review Article by Feyder et al. (2011), which provides an update of the mechanisms implicated in L-DOPA-induced dyskinesia, with particular focus on abnormal signaling processes affecting the cAMP and ERK cascades and occurring in dopamine D1 receptor expressing striatal neurons.

The dopamine- and cAMP-regulated phosphoprotein Mr 32,000, DARPP-32, is a key signaling molecule necessary for a correct functioning of the striatum and basal ganglia. Yger and Girault (2011) offer a critical appraisal of the molecular properties of this important signaling component, focusing on the regulation by various classes of psychoactive drugs and on the involvement in neuropsychiatric diseases.

The mammalian target of rapamycin (mTOR), is a fundamental regulator of mRNA translation and has been implicated in a plethora of physiological and pathological conditions. This subject is addressed by Santini and Klann (2011) who describe the molecular mechanisms at the basis of mTOR-mediated control of *de novo* protein synthesis and provide a number of examples of how perturbations of this signaling cascade represent a common pathophysiological feature of neurodevelopmental, neuropsychiatric, and neurodegenerative disorders.

We hope that the reader will enjoy this collection of articles and find it useful as a source of information on a number of specific aspects related to a broad subject of study. We are very grateful to the many contributors and to all the colleagues involved in the reviewing process for having made this endeavor possible.
